# Treatment of Typhoid Fever by Mercurials

**Published:** 1851-02

**Authors:** 


*Treatment of Typhoid Fever by Mercurials.* M. Becquerel has lately submitted to the French Institute a memoir on the treatment of Typhoid Fever by Mercurials, which is well worthy of attention.

In the year 1847, M. Serres read at the Institute a series of papers on "The Nature and Treatment of Typhoid Fever," and proposed a new mode of treatment, which he considered likely to exercise a most beneficial influence on the progress and character of that disease. The mercurial treatment of M. Serres consisted in the internal use of the black sulphuret, in doses varying from twelve to sixty grains, and in frictions over the abdomen with common mercurial ointment, in quantities varying from half an ounce to one ounce daily.

This treatment was continued for ten or twelve days—even longer, until the prominent symptoms of the fever had given way.

Having been appointed to take charge of M. Serres' patients at la Pitié for some time, M. Becquerel resolved on submitting the mercurial method to further experiment. He therefore made trial of it on fifteen patients affected with typhoid fever, abstaining, with some slight exceptions, from the use of any other remedy.

The black sulphuret of mercury was administered to each patient immediately on his admission; he began with thirty-two grains, in the form of pill or powder, divided into five or six doses. If no improvement took place in two or three days, the dose was increased to forty-eight or even sixty-two grains. As soon as convalescence commenced, the remedy was discontinued. In no case was the salivation severe enough to render discontinuance of the mercury necessary.

The mercurial ointment was employed in the quantities recommended by M. Serres, being increased in such cases as demanded an increase of sulphuret. Thus, with thirty-two grains of the sulphuret, sixteen scruples of mercurial ointment were employed in two frictions; and with sixty-four grains, thirty scruples. In order to favor absorption, the abdomen was cleansed every day with soap.

The accessory remedies employed were—ice, seltzer-water, and lemonade, as a drink; simple lavements, or, in cases of constipation, a laxative clyster.

In ataxic cases, of which four occurred, musk was administered as a stimulant to the extent of five or six grains a day.

The following are the results of the practice:—Fifteen patients, all laboring under typhoid fever in a severe form, were treated—ten men, five women. The men were aged—2, 16 years; 2, 17; 2, 18; 1, 20; 2, 22; 1, 36 years. The ages of the women were—15, 18, 20, and two of 21 years.

The male cases may be arranged as follow: 4 laboring under the ordinary abdominal form; 5 presenting adynamic symptoms of a very severe nature; and 1 with ataxic fever, in which the delirium and agitation predominated.

Of the females, 1 was affected with the adynamic form; 1 with common typhoid fever; 1 with the ataxo-adynamic, and two with the ataxic fever. Dating the commencement of the attack from the first appearance of a febrile movement, we find that 2 entered hospital on the second day; 2 on the fourth; 7 on the fifth; 3 on the sixth; and 1 on the eighth day. The premonitory symptoms of headache, muscular depression, &c., had, besides, varied from three to twelve days before the regular febrile attack commenced.

The treatment was begun, in the 15 cases, on the day after admission. The following were its principal effects:—

*Febrile Movement.*—The first few doses of the sulphuret, and a few frictions, diminished the heat and dryness of the skin, which in some cases became moist. The pulse, at the same time, constantly fell. It varied from 84 to 116 in the different cases, and in all, the frequency was diminished by the third day.

*Mouth.*—The dry, rough condition of the tongue, and the blackness of the lips, never disappeared until salivation had set in. The latter occurred twelve times in the 15 cases: it was absent twice; and in these two cases, recovery was just as prompt as in the former. In the only fatal case, the tongue remained dry to the end of the disease. In every case but one the salivation was slight: in the one excepted, the tongue and gums continued swollen for twelve days.

With respect to this effect, the following propositions appear to be established:—

1. In ordinary typhoid fever of moderate intensity, salivation occurs more quickly than in the other forms; it is more decided, and continues a little longer during the convalescence, of which it is usually the precursor.

2. In severer cases, the salivation occurs at a later period, and is less considerable; sometimes it takes place at the moment the febrile symptoms disappear; in other cases, a few days previously.

3. In the most dangerous cases, it is very difficult to excite salivation, and, until this takes place, we have always to dread some accident. Great perseverance is therefore requisite.

4. Finally, in a few mild cases, salivation does not exist, the mercury appearing to master the disease in less doses than are necessary to stimulate the salivary glands.

*Tympanitis.*—In every case, except the fatal one, this symptom diminished with great rapidity, and that from the commencement of the treatment.

*Bowels.*—The mercury produced, in two cases, one or two fluid stools daily; in two others, accompanied by constipation, it had no laxative effect, and lavements became necessary. In three cases of diarrhœa (five or six fluid stools a day) the looseness diminished in a remarkable manner as soon as the remedy was employed. In eight cases it had no effect whatever on the state of the bowels, neither augmenting nor diminishing the diarrhœa.

*Rose Spots.*—These spots, when situate on the abdomen, were invariably removed in twenty-four to thirty-six hours by the mercurial frictions; but they continued their course on the other parts to which mercury was not applied.

*Delirium.*—When this symptom was at all prominent (four cases) musk was given to the extent of five or six grains daily. Although so violent that the use of the straight-jacket had been necessary, these patients became calm in four or five days. As to the cough, chest symptoms, and stupor, they followed the general course of the disease, and appear to have been modified in a secondary way only.

*Duration of Treatment.*—In four cases the treatment was prolonged to 7 days; in three cases, to 8 days; in one to 9 days; in three to 10 days; in one to 12; in one to 15; in one to 16; in one to 17 days. The mean duration was, therefore, 10 days.

The quantity of mercury employed was, on an average, 12½ scruples of the sulphuret, and 200 scruples of mercurial ointment. The smallest quantity was, 7 scruples of the sulphuret, with 112 scruples of ointment; the maximum, 24 scruples of the former and 360 of the latter.

*Duration of the Disease.*—The mean duration of the disease in fifteen cases was 16 days. In one case the duration of the fever was 12 days; in two, 13 days; in three, 14; in three, 15; in one, 16; in one, 18; in two, 20 days; in one, 21 days; and in one, 23.

From the above facts M. Becquerel draws the following conclusions:—

The treatment of typhoid fever by the black sulphuret of mercury and mercurial frictions over the abdomen, is attended by the most advantageous results. The effects are more striking in proportion as the disease is treated at an early period. In fifteen cases, all of a severe nature, taken indiscriminately, only one death occurred, and that from perforation of the intestine. The duration of the disease, also, was unquestionably shortened by this method of treatment, for it varied from twelve to twenty-three days. The duration of the treatment varied from seven to seventeen days.

The following were the most remarkable effects of the remedy:—Diminution of the force and frequency of the pulse, and of the heat of skin; *rapid and early disappearance of the typhoid eruption*; rapid removal, also, of the tympanitis; excitement in a great majority of the cases of salivation on the 6th to the 13th day, which is almost a certain sign that the treatment has succeeded, and that the patient will recover. Combined with mercury, the musk was effectual in subduing rapidly the most severe ataxo-adynamic symptoms.

Since the publication of M. Becquerel's memoir, ten additional patients, laboring under severe typhoid fever, have been cured by the mercurial method. This gives a sum of twenty-five cases, with one death—a result which certainly entitles the practice to consideration, if it does not at once stamp its merit.—*London Medical Times*, Nov. 2, 1850.

